# The Predecessors of Bitcoin and Their Implications for the Prospect of Virtual Currencies

**DOI:** 10.1371/journal.pone.0123071

**Published:** 2015-04-28

**Authors:** Thomas Kim

**Affiliations:** School of Business Administration, University of California Riverside, Riverside, CA, 92521, United States of America; Wenzhou University, CHINA

## Abstract

To examine whether the recent price patterns and transaction costs of Bitcoin represent a general characteristic of decentralized virtual currencies, we analyze virtual currencies in online games that have been voluntarily managed by individuals since 1990s. We find that matured game currencies have price stability similar to that of small size equities or gold, and their transaction costs are sometimes lower than real currencies. Assuming that virtual currencies with a longer history can provide an estimate for Bitcoin’s prospects, we project that Bitcoin will be less influenced by speculative trades and become a low cost alternative to real currencies.

## Introduction

Throughout history, the management of currency has been a responsibility and a right of a central authority. The recent debut of virtual currencies, such as Bitcoin, however, challenges this tradition. Electronic currencies are unique as the creation and management of these currencies are done by non-government entities. Further, there is an ongoing debate regarding whether these decentralized currencies are capable of serving as a substitute for the role of real currencies. In his letter to the Senate in 2013, Bernanke, the Federal Reserve Board chairman, states that cyber currencies “may have long-term promise.” On the other hand, Krugman [1] criticizes virtual currencies because they are used primarily for speculation rather than as a method of transaction.

It has been only a few years since Bitcoin received much public attention, and it is difficult to determine whether the current observations from Bitcoin represent general characteristics of decentralized virtual currencies or are Bitcoin-specific. To acquire a more general understanding about virtual currencies, we examine a similar type of virtual currency that has been voluntarily traded and managed by individuals since the 1990s. The first cash payments between players for a virtual item occurred in 1987 (Heeks [2]). The purpose of our analysis is to obtain the prospects of Bitcoin from other similar virtual currencies with a longer history.

Multiplayer online games have their own virtual currency to facilitate in-game transactions among game players. The virtual currencies used in online games closely resemble the virtual currencies recently developed for transaction purposes, including Bitcoin, in that the currencies are created and managed by non-government entities and are based on cyber space. Game currencies have surprisingly large economic significance. Korea Bureau of Statistics estimates that the annual dollar volume of game currency trading is over $1 billion in 2007 in Korea alone and that the volume is growing. Mt. Gox exchange, which handled 70% of the Bitcoin transactions worldwide in 2013, was initially an exchange trading online game related items. Many of these game currencies have been used for more than a decade, and the number of currency users is sometimes larger than a nation’s population. For example, *World of Warcraft*, an online game, has more than 7 million players. The game servicing company, Activision Blizzard, reports 7.6 million subscribers as of the second quarter of 2014.

Our empirical analyses are focused on two aspects that Bitcoin has been criticized for. The first refers to high price volatility. To be a used as money, a currency should not have too much volatility as money has to be a unit of account or store value (Yermack [3]). Yermack [3] argues that Bitcoin prices are driven by speculative factors, based on the observation that Bitcoin prices have high volatility and almost no correlation with other assets. A possible basis for this high volatility is the absence of a central authority. This paper examines whether game currencies, which are also managed by non-government entities, display similar characteristics.

The second aspect concerns ambiguous transaction costs. Although enthusiasts insist that Bitcoin can provide lower transaction costs than real currencies, there are few empirical tests to support this argument. This paper investigates whether the transaction costs of virtual currencies can be lower than that of real currencies. Instead of relying on theoretical arguments about the transaction costs, we calculate the costs directly from our empirical price data.

We find that the recent high volatility of Bitcoin is not a general characteristic of a non-government managed virtual currency. While new game currencies are as volatile as Bitcoin, matured game currencies have one-third of Bitcoin’s volatility, a level similar to that of a small size equity listed on a U.S. public exchange. Economic factors, such as exchange rates or game servicing a company’s stock returns, explain 40% of all game currency price variations indicating that the prices are not totally determined by speculative factors.

Transaction costs are calculated with two measures endorsed by the market microstructure literature. The negative covariance measure of Roll [4] and the Zeros measure of Lesmond, Ogden, and Trzcinka [5] demonstrate that the transaction costs of game currencies and Bitcoin are sometimes lower than that of real currencies, such as the Euro. The transaction costs of a virtual currency significantly differ by the degree of competition among the exchanges. Country specific regulations, such as residency checks, seem to protect inefficient exchanges.

Assuming that the case of more matured game currencies indicate Bitcoin’s prospects, we project that Bitcoin will have lower price volatilities and higher correlations with economic factors. If Bitcoin can combine the price stability with its already low transaction costs, it may become a more widely accepted method of transaction.

## Virtual Currencies in Online Games

### Creation and Management of Virtual Currencies in Games

Game currencies are virtual currencies used in multiplayer online games. Multiplayer online games are a type of computer game that multiple users play together over the Internet. A typical form of these online games is a genre known as “Massively Multiplayer Online Role Playing Game” (MMORPG). The genre has two important characteristics. First, each player has to create a cyber character called an “avatar.” Additionally, there is interaction, such as battles, among different players’ avatars. These two characteristics encourage players to compete with each other in developing their avatars. A more powerful avatar in a game provides the personal utility of winning a competition and enjoying more game content. These cyber competitions may be directly related to real rewards. A few online games allow powerful players to collect taxes from other players, and the collected game currency can be converted to real currency in the game currency markets.

Avatars become more capable and powerful when: 1) players spend more time on the game and 2) players equip the avatars with better items. It is in the equipment that real world wealth can make a large difference. Players are willing to spend real wealth to acquire a scarce virtual item so that they can gain more utility from a game.

The competition for game items and the willingness to spend real world wealth gave birth to markets in which players can purchase game items with real currency. Markets for cyber items are typically separate websites where players can trade game goods (items and cyber currency) with each other. These markets connect the game currencies with the real economy, as game currencies are priced in terms of real currencies.

There is a considerable demand for these markets. However, an obstacle to cyber goods trading is that the transfer of cyber goods and the payment of real currency cannot occur simultaneously. The payment of real currency can be made by direct wire transfers or payment services, such as PayPal. The transfer of cyber goods, in comparison, must be made within a game. A seller and a buyer should both have an avatar in the game, and the seller should transfer the cyber goods from their avatar to the buyer’s avatar. Because it is cumbersome to trade every item in two different spaces, players use game currencies as a method of transaction. Each online game has its own virtual currency that is used to trade goods and items in the game. The in-game transaction process is comparable to a real world version of giving a dollar bill to a person for a good or service. In most of the games, it is as simple as sending or receiving emails. Players typically convert real currency into game currency using outside markets and then complete most of game-related transactions with game currency. They can convert a game currency back to a real currency in the game currency markets. In virtual worlds, game currencies are a better method of completing a transaction than are real currencies. Game currencies are similar to Bitcoin in that both currencies are created in a virtual world and can be converted to real currencies in specified markets.

Game currencies share another important feature with Bitcoin; that is, the currency can be created by the players. Players can create game currency by completing a task in a game. For example, a player may earn one piece of game currency by hunting down a deer. The hunting creates additional game currency in the game economy as new currency is added to the game economy and a virtual deer (no value) disappears. Alternatively, a player can spend the currency to purchase game goods for his avatar. The game currency is effectively erased from a game economy when a player spends the currency to buy consumable goods from a game, such as food for his avatar. Thus, the creation and consumption of cyber currency in online games is largely influenced by game players.

Since game currency can be created by the players and markets for the game currency enable players to exchange cyber goods for real currency, there are those players who play only to create game currency and to sell it in markets for real money. These players repeat tedious tasks, such as hunting a deer, to accumulate game gold. They may get $10 by selling their 1,000 pieces of game gold at markets for game currency. This type of game currency generation process is often executed on an industry scale. Dibbell [6] reports that as of 2007, the size of this “gold mining” industry was $1.8 billion. The massive creation of cyber money is problematic to a game’s economy, as it will generate higher inflation of the cyber currency. In response, game managers often have a dedicated team to ban these systematic activities of game currency creation and to develop game contents that force players to spend more game currencies. Game players also cooperate to with the game managers’ policies as they recognize a greater inflation of the currency is harmful to their wealth in cyber space.

The issue of industrialized game currency creation closely resembles what is recently occurring in Bitcoin mining. With the recent public interest in Bitcoin, the Bitcoin mining process is becoming more industrialized. Since a better computing system has a greater probability of solving the math challenges of Bitcoin, miners who can pool more computing resources are likely to acquire more Bitcoins. Unlike game currencies, however, Bitcoin does not yet have a mechanism to control the amount of rewards for industrial mining.

### A Comparison Between Game Currency and Other Virtual Currency

A comparison between game currency and the recently debuted virtual currencies would be useful in understanding the general characteristics of virtual currencies. A virtual currency is comprised of digital data that can be used as a method of transaction among Internet users. The most important feature of virtual currencies is that non-government entities can create or manage the currencies. A game player can create game currency through various game activities. Bitcoin is created through a computer system that solves a decoding problem.

These free creation mechanisms can create supply problems like high inflation. It is said that Bitcoin does not have these supply related issues as the supply is controlled by a math algorithm. The maximum number of Bitcoins is 21 million (Surowiecki [7]) and the algorithm of the Bitcoin system is designed to generate a pre-determined rate of Bitcoins. However, the structure of Bitcoin is not absolutely fixed. Bitcoin users can implement new changes to the structure if a majority of the users agree to do so. A shock from outside of the system can also change the structure. For example, the rate of the Bitcoin supply is determined by the balance between the difficulty of the encryption and the decoding power of today’s computers. If computing power improves substantially, the math algorithm may not be able to maintain the projected speed. Bitcoin wiki website (en.bitcoin.it) already worries about the possible debut of quantum computers, which can solve the current math exercise of Bitcoin at a much faster rate. In addition, there is the possibility that the Bitcoin system could be hacked. Although the system is regarded as secure at this point, without a central authority that has enforcement power, this currency may be facing attempts by outsiders to break the system.

Alternatively, individuals who are already holding a significant amount of virtual currency have an incentive to protect the value of this currency. Voluntary regulation already takes place in game currencies. Anecdotal evidence indicates that game players voluntarily regulate industrialized currency creation attempts, knowing that industry level mining generates inflation in the cyber economy. For example, in a game that allows fighting among players, players attack other players who are suspected of game currency mining. The attack interferes with the mining activities executed by automated programs. Game players even pressure game servicing companies to maintain their virtual currency value. For example, game players pressure the servicing companies to provide safety mechanisms for their currency values, such as adopting anti-hacking software. This pressure may come from non-virtual methods including demonstrations or even law suits. In a lawsuit on the trading of game currencies, South Korea’s Supreme Court decided that “cyber goods are a result of one’s effort and should not be taken away from the owner without a significant reason,” acknowledging that cyber goods may have a real value that should be protected by law. These individual incentives may be the strongest mechanism to control the value of a non-government managed virtual currency. Accordingly, even if game servicing companies have considerable control over game currencies, they cannot easily impose radical “monetary policies,” such as shutting down the game and voiding the currencies. If a game is to be shut down, in most cases, the game currency is converted to the currency of another game managed by the same servicing company. It would be comparable to a large shock to real currencies provoking public unrest. Bitcoin users also have incentives to guard their currency value, and they may display actions similar to that of game currency holders.

The other important characteristic of virtual currencies is anonymity. Real money transactions conducted through the Internet are closely monitored and recorded by the government and by financial institutions. In contrast, since virtual currencies are managed by individuals, there is no designated monitor of the currencies. Game currencies and Bitcoin share a similar degree of anonymity. One can trade Bitcoin anonymously, as the transaction record does not completely reveal the true identification of a trader. It indicates only that a user who has cyberspace name “xy23z” used a certain amount of Bitcoin. Bitcoin users can generate billions of Bitcoin trading accounts (called “wallets”) and it is difficult to connect each of these accounts with a real identity. Due to its anonymity, there is the suspicion that Bitcoin is used for money laundering (FBI Report [8]).

Unlike Bitcoin, transaction records for game currencies are not automatically generated. The transactions can be recorded by game managers for back-up purposes, but those managers have little incentive to keep the records for an extended period of time as it requires the dedication of too many resources to maintain such a database. As a result, it would be difficult for a third party to track the whole transaction. In a similar vein, the New York Times reports that the NSA and CIA have been secretly trying to monitor in-game activities since 2008 for the purpose of discovering illegal activities (Elliott [9]). It can be said that anonymity reduces the costs of the transactions that are regulated by law, such as trades related to illegal goods, tax evasion, or money laundering.

Overall, game currencies and Bitcoin share two important characteristics of a virtual currency: 1) the management by individuals and 2) anonymity in cyber space. Thus, by examining game currencies, we expect to acquire an estimate of the prospects of Bitcoin and other recently debuted virtual currencies.

## Empirical Analysis on Game Currency

### Game Currency Data

South Korea is known to have the largest market of gaming goods trading and the most detailed gaming goods transaction statistics in the world (Heeks [1]). The price data of game currencies in this paper are acquired from, www.itemmania.com, one of the two largest online markets for gaming goods in the country. The two largest markets comprise over 90% of the market share in South Korea, and both of the markets are indirectly owned by a U.S. investment bank, Goldman Sachs. As a result, the structures of the two markets are similar to each other. The market structure resembles that of a stock exchange; trades occur continuously and traders can place either a limit order (place quotes) or a market order (accept the existing quotes). The market provides daily game money price data. The data defines a daily price as the last trading price near midnight, as this market operates 24 hours per day.

We also obtain price data from a U.S.-based cyber goods trading website, www.ige.com. The website is closer to a dealer than to a market in that players cannot post their quotes and can only accept the website’s quotes. The website is known as one of the most frequently used gaming goods trading websites in the U.S. The site revises its quotes intermittently, approximately once a week. The site displays only sell (ask) quotes to the public.

It is worthwhile to note that the primary position of these markets and dealers is to keep the transaction information private. As such, it is difficult to collect data concerning game currency trades. This secrecy is related to several factors. First, many game players are not comfortable with other players’ using real wealth to win competitions in a game. Buying gaming goods with real currency is sometimes regarded as “cheating.” The markets and their participants seek to hide their transactions to dodge the criticism of other players. Additionally, game managers have not reached a consensus regarding the real money trading of their game goods. Heeks [1] reports that the reaction of game managers to cyber goods trading varies from an attempt to completely ban it to a laissez-faire policy. The policy may also change over time in the same game. A game goods trader may receive a penalty from game managers unexpectedly. Moreover, legal authorities have made several attempts to regulate cyber goods trading when they suspect money laundering activities or fraud. Similar to game managers, regulators do not have an organized view of cyber goods trading yet making regulation unpredictable. For example, South Korea’s Supreme Court decided that the “trading of cyber goods is legal” in 2009, while in 2013, the Korean Ministry of Culture, Sports, and Tourism attempted to ban all cyber goods trading in non-adult-rated games. Overall, there are plenty of incentives to conceal the transaction data. Accordingly, we made the name of the Korean game currency market and the U.S. website anonymous in this paper to prevent any issues in regard to the use of publicly posted price information. Details regarding the data sources are available upon request.

This study employs the game currency prices of the games *Diablo III* and *World of Warcraft*. While the game currency trading data are from South Korea, *Diablo III* and *World of Warcraft* are made and managed by Activision Blizzard, a U.S. company, and the games are played worldwide. Therefore, the two game currencies are less influenced by country-specific factors. We acquire historical prices of the two game currencies. In the market from which we obtain the data, historical prices are available for only a handful of the most widely traded game currencies.

*World of Warcraft* is the most popular online game in the world and holds the Guinness World Record for the largest number of subscribers around the globe (10 million subscribers in 2008). As a result, its game currency is the most widely traded game currency in the world. The history of *World of Warcraft* game currency trading spans over a decade. *Diablo III* has over one million players daily. It is a fairly new game, released in May 2012. The game is particularly interesting in that it has its own market for cyber goods called “Real Money Auction House” (RMAH) that allows a player to buy *Diablo III* items from other players with real currency, such as U.S. dollars. A player can use a real currency that is convenient for that player. For example, a Canadian user may use Canadian dollars, while a Russian user may use Rubles. The establishment of RMAH was a response from the game servicing company, as many users have already been trading game currency with real currency.

From the Korean market, we acquire about one year of daily prices of the *Diablo III* game currency from November 2012 to November 2013. In addition, approximately five months of *World of Warcraft* game currency prices are accessible (from July 2013 to November 2013). The price data from the U.S. dealer is scarcer. About two months of price data is available per game. Although the *World of Warcraft* pricing data have shorter time-series coverage, the data are much broader cross-sectionally as the data contain prices from 12 servers as compared to only three servers for *Diablo III*.

**Fig 1:**
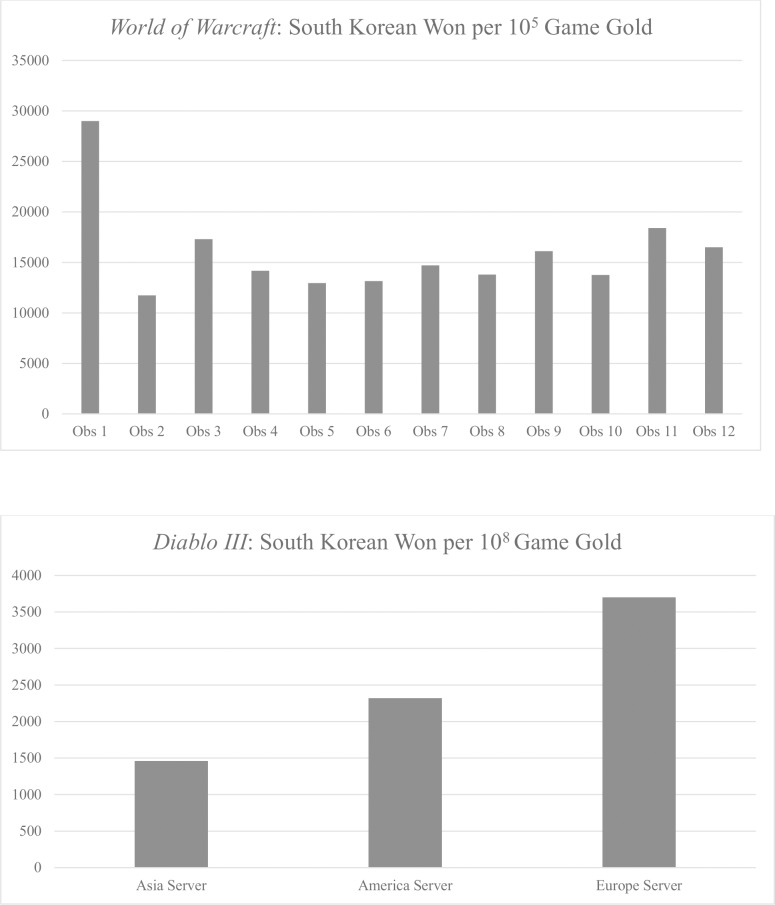
Price Distribution by Different Servers and Sides. This figure illustrates average game currency prices in South Korean Won. Each bar represents an observation from a server/side. For *World of Warcraft*, there are six servers in our dataset and each server has two sides for a total of 12 observations. *Diablo III* has three servers without sides for a total of three observations.

Even in the same game, the currency prices vary by server. Online games are separated into smaller units of servers. A server is a computer system that connects multiple players. Due to capacity restraints, online games must have multiple servers. The amount of data that a server can process increases exponentially with the number of players on a server, as one player’s actions could create feedback from many other players. To facilitate game management, game providers allow little data sharing between servers. As such, a player in Server A cannot interact or participate in a transaction with a player in Server B within the same game. Likewise, game currency in one server cannot be transferred to another server. Due to the isolation of servers and because each server will have a different number of players, game play styles, and so forth, the value of one server’s currency can be quite different from the value of another server’s currency. As of 2013, there are approximately 1,000 *World of Warcraft* servers around the globe, but price data are available for only six of the servers that are played primarily by South Korean users. Fig 1 illustrates the distribution of cyber currency prices of *World of Warcraft* servers and *Diablo III* servers as of October 31, 2013.

We obtain price data for six servers of *World of Warcraft*, but there are 12 prices in Fig 1 because even within the same server, the price differs by the side a gamer is playing. In *World of Warcraft*, a player has to select a side to play (Alliance vs. Horde) and cannot switch his avatar’s side once the choice has been made. Further, no direct transaction is allowed between different side players. As such, there are a considerable number of transaction costs involved to transfer a good from one side to the other. The different prices are actually a sign of efficiency. Whenever there is a restriction in currency flow, an efficient market should have different prices for each isolated economy so that the prices reflect the unique economic situation of each cyber economy.

### Price Stability

The decentralized control structure of virtual currencies may generate an irrational pattern or high volatility in their prices. This view is supported by the recent high volatility of Bitcoin. Our first analysis is to examine if these high volatilities are Bitcoin specific or one of the characteristics of non-government managed virtual currencies. We compare the price volatilities of non-government managed virtual currencies (game currencies and Bitcoin) with that of real currencies and frequently traded assets. Note that game currencies do not have a supply controlling algorithm such as Bitcoin’s. This difference may generate higher volatilities in game currency prices.

Using the price data obtained from the South Korean exchange, we calculate the daily mean and standard deviation of game currency price changes by each server/side and report the average of the statistics. We also present the annualized volatility calculated from the daily standard deviations. Following standard procedures, the price change of a currency is defined as the difference between two consecutive days’ prices divided by the previous day’s price.

$${\text{Return}}_{i,t}=\frac{Price_{i,t}-Price_{i,t-1}}{Price_{i,t-1}}$$ (1)

For comparison, we display the returns of Bitcoin, Euro, Japanese Yen (JPY), South Korean Won (KRW), Chinese Yuan (CNY) exchange rates with U.S. dollars, gold, and stock prices in 2013. Other than the Euro, JPY, KRW, and CNY are the Asian currencies where most of virtual currency trading occurs. The Mt. Gox exchange, a Japanese exchange, handled 70% of the worldwide Bitcoin transactions in 2013. China and South Korea also have large markets for game currencies, as well as for Bitcoin. Bitcoin prices are obtained from www.coindesk.com and the exchange rates of real currencies are acquired from www.oanda.com. Gold price data comes from the Federal Reserve Bank of St. Louis website. Stock prices are acquired from the Center for Research in Security Prices (CRSP) at the University of Chicago. The stocks are grouped into three size categories, small, medium, and large, according to their market value at the beginning of 2013. Each business day, we calculate the cross-sectional statistics of stock returns, such as the mean or the standard deviation, and then compute the year average of the daily statistics. Table 1 reports the summary statistics.

**Table 1:** Summary Statistics on Price Changes.

Currencies or Assets	Mean of Daily Returns	Standard Deviation of Daily Returns	Annualized Volatility
*Diablo III*	-0.2%	9.3%	146.9%
*World of Warcraft*	0.5%	2.7%	42.7%
*Bitcoin*	1.4%	7.3%	115.3%
USD per 1 Euro	-0.0%	0.4%	6.3%
USD per 1 JPY	0.1%	0.5%	7.9%
USD per 1 KRW	0.0%	0.8%	12.6%
USD per 1 CNY	-0.0%	0.1%	1.6%
Gold Prices	-0.1%	1.3%	20.5%
Equities(Small Size)	0.0%	2.9%	45.8%
Equities(Middle Size)	0.0%	2.1%	33.2%
Equities(Large Size)	0.0%	1.6%	25.3%

We report summary statistics on the daily returns of various assets. The mean, standard deviation, and annualized volatility are reported. The data period is from November 2012 to November 2013 for *Diablo III* currency and from July 2013 to November 2013 for *World of Warcraft* currency. For comparison, we report the statistics of Bitcoin, Euro, Japanese Yen (JPY), South Korean Won (KRW), and Chinese Yuan (CNY) exchange rates with the U.S. Dollar, as well as gold and equities. We use all of the daily prices in 2013 for the assets not related to games.

The standard deviation of game currency returns is 9.3% for *Diablo III* currencies and 2.7% for *World of Warcraft* currencies. Bitcoin’s standard deviation is 7.3% during the entire year of 2013. These figures are higher than any of the real currency exchange rates reported, indicating that virtual currencies have higher volatilities than real currencies. The annualized volatilities of the *Diablo III* currency and the *World of Warcraft* currency are 146% and 43%, respectively. The price volatility of the *Diablo III* currency is 13% higher than that of Bitcoin, while the volatility of the *World of Warcraft* currency is about one-third of Bitcoin’s and similar to that of small or middle sized equities. The question is what level of volatility is acceptable as a currency. Since equities are sometimes used to store value, we suppose that the volatility of the *World of Warcraft* currency is at a level enabling the currency to operate as an (inferior) alternative of a real currency. Also, equities held by a firm are often regarded as a cash equivalent asset in accounting. Note that *Diablo III* began its service only in 2011, while *World of Warcraft* has a history longer than a decade. Bitcoin gained much of its current public attention around the time when *Diablo III* was initiated. The case of the *World of Warcraft* currency demonstrates that the high volatility of Bitcoin is not a common attribute of non-government managed virtual currencies. Rather, high volatilities may be a characteristic of newly introduced currencies, as demonstrated in the case of *Diablo III* currency.

An alternative way to measure price stability is to examine the size of devaluation or revaluation during a certain period. Too much devaluation or revaluation is an indication of poor stability. Devaluation can be particularly problematic to game currencies as any player can create the currencies by playing a game. Alternatively, the math algorithm of Bitcoin is designed to reward new Bitcoin to a few miners at a pre-determined rate.

The devaluation of a currency is calculated by (the price at the beginning/the price at the end—1). Positive numbers represent devaluation (decrease in value), while negative numbers represent revaluation (increase in value). The prices are measured in U.S. dollars. Table 2 shows the devaluation of currencies.

**Table 2:** Devaluation of Currencies.

Currencies or Assets	Devaluation = (price at the beginning / price at the end—1)
*Diablo III*	1,378.2%
*World of Warcraft*	-34.1%
*Bitcoin*	-98.2%
Euro	4.3%
Japanese Yen	-18.1%
South Korean Won	0.8%
Chinese Yuan	3.2%
Gold	40.0%

We measure the devaluations of various currencies and gold. The devaluation of a currency is calculated as: (price at the beginning/price at the end—1). A negative devaluation indicates an increase in currency value (revaluation). Currency prices are measured in U.S. dollars, using the exchange rates acquired from www.oanda.com. The data period is from November 2012 to November 2013 for *Diablo III* currency and from July 2013 to November 2013 for *World of Warcraft* currency. We use the full 2013 data for Bitcoin, real currencies, and gold.

*Diablo III* has a devaluation of 1,378% during a one-year period, while the *World of Warcraft* currency price increased by 34%. It is actually surprising to observe a revaluation of a game currency. Since the currency can be created by individuals, the revaluation indicates that there are effective mechanisms that can offset/overcome the continuous mining activities of some individuals. These mechanisms include an increased amount of in-game spending and the supervision of mining activities. Anecdotal evidence suggests that not only game managers, but also game players supervise each other to prevent losses in game currency value. The case of *Diablo III* may indicate that the free creation of game currencies leads to the devaluation of the currency, but the case of *World of Warcraft* stands as a counter example. Bitcoin has a price increase of 98%, which is a substantial increase in the value. Real currencies have devaluation or revaluation rates under 20%. Again, the subsequent question is what devaluation rate is to be regarded as too large. We suppose that the change in gold prices can serve as a basis. Gold, an asset that is sometimes used in lieu of real currencies, has a 40% devaluation in 2013. *World of Warcraft* currency has a smaller price change (in absolute value) than gold. Thus, we find that the price stability of some virtual currencies is comparable to that of frequently traded assets like gold or small size equities.

The instability and large devaluation of *Diablo III* currency is a typical pattern of virtual currencies in newly launched online games. Inflation is inevitable in new games as: 1) the liquidity of game currency must be increased rapidly to accommodate new incoming players and 2) the degree of competition among players is typically greatest at the beginning of a game. This is because a new game is like a new land where a player can obtain an advantage over others if they become the first to possess the land. Game currency prices are the highest at the beginning as players compete to be the first. The price quickly declines as a game economy becomes more established. Similarly, Bitcoin may have large price volatility because it is a newly launched currency.

### Price Determinants

Another uncertainty concerning Bitcoin arises in that Bitcoin prices have almost no correlation with other assets (Yermack [3]). We examine whether this is true for other virtual currencies as well. We use a multivariate regression to test the relationship between virtual currency prices and economic factors. This analysis is feasible for game currencies as our data provides a few variables possibly related to game currency prices. This analysis would be one of the first attempts to identify the factors that determine the price of a virtual currency.

We examine the supply and demand factors of the game currency used in *World of Warcraft*. We have price data for 12 of its game servers and sides, as well as some information about the server characteristics. As a supply factor, we estimate the number of professional game currency creators, also referred to as miners, by server/side. The number can be estimated from the quotes on the market for game currency. Every week, we compute the number of sell quotes per *World of Warcraft* server that are posted within the previous three hours. Professional currency miners try to sell their game currency through the markets quickly, and they update their quotes at least every hour for better visibility. Individual sellers, in contrast, do not update their quotes as frequently, and their quotes are often older than one day. Therefore, a higher number of fresh updated sell quotes indicates a greater number of professional currency miners.

We measure the demand for game currency with two variables. The first demand variable is the number of players in each server. There would be more demand for game currency when there are more players. The cross-sectional difference in the number of players can be estimated by the server status report on the *World of Warcraft* website. The website periodically provides information on the crowdedness of a server using a three-step indicator (low, medium, and high). In the regression analysis, we use a three-step variable that takes a value from one (Low) to three (High) to proxy for the number of players by server.

The next demand factor is the degree of competition. Players try to win the in-game competition by better equipping their avatars. This competition leads to more game related trading, which increases the demand for game currency. The degree of competition can be captured by server type. The servers of *World of Warcraft* have slightly different game contents in terms of competition. A Player vs. Environment (PvE) server allows battles between players, but the battle must be consensual. In a Player vs. Player (PvP) server, a player can be attacked by an opposing side player at any time. Therefore, the level of competition among players can be higher in a PvP server. We use a dummy variable for server type that takes a value of one when a server is a PvP server and zero otherwise. Both of demand variables are collected every week.

As a control variable, we include real currency exchange rates. The price of a game currency is in South Korean Won (KRW), as the market is mainly for South Korean players, but the game’s currency is also used by players outside of the country. Thus, a devaluation of the South Korean Won may be accompanied with a revaluation of a game’s currency. Daily exchange rates between South Korean Won and U.S. dollars are obtained from quotes from the Korea Exchange Bank. This exchange rate represents the retail exchange rate (the rate that banks charge to retail customers) in South Korea.

Another control variable is the stock returns of the game management company, Activision Blizzard. This variable can represent other supply and demand factors for their game currency, as stock prices are likely to be correlated with the contents of the game. According to the firm’s 2010 annual report, over 80% of the firm’s revenue is related to *World of Warcraft*.

The explanatory variables do not have enough time-series variability when compared to game currency prices. The most frequent series are exchange rates and stock returns, but they are not available on non-business days. Other variables, such as the number of quotes or server types, are obtained weekly. Alternatively, game currency prices are available seven days a week. We use weekly game currency prices that are measured every Friday. Likewise, weekly stock returns and exchange rate changes are calculated from Friday prices.

Our left-hand side variable is the weekly game currency prices, while our right-hand side variables are the number of currency creators measured by the number of fresh quotes, server crowdedness, server competition, KRW/USD exchange rate changes, and the game manager stock returns. The regression utilizes panel data that pools prices from different *World of Warcraft* servers/sides. We include server dummies (one if it is Server A and zero otherwise) to correct for server fixed effects. The estimation equation has the following form:

$$\begin{array}{l} Log(Price_{i,t})=\alpha +\delta_{1}\cdot Quotes_{i,t}+\delta_{2}\cdot Server\;Crowdedness_{i,t}+\delta_{3}\cdot Server\;Competition_{i,t}\\ \;+\delta_{4}\cdot Exchange\;Rate_{i,t}+\delta_{5}\cdot Stock\;Returns_{i,t}+\varepsilon \end{array}$$ (2)

where Log(*Price*_*i*,*t*_)is the natural log of game currency price in server or side *i* at week *t*, *Quotes* is the number of fresh quotes on the market for game currency, *Server Crowdedness* is the number of users on a server measured by a three-step scale (high, medium, and low), *Server Competition* is a dummy variable that is equal to one when a server has more competition among players, *Exchange Rate* is the exchange rate between KRW and USD, and *Stock Returns* is the weekly stock returns of the game servicing company. The estimation method is Generalized Method of Moments (GMM) that corrects for heteroscedasticity and autocorrelation in the regression error terms. Table 3 presents the results of the regression.

**Table 3:** Game Currency Price Determinants.

Dependent Variable:Game Currency Price by Server/Side in KRW	Full Model	Game Related Variables Only	Financial or Economic Factors Only
Number of Sell Quotes	-0.04^a^(-4.80)	-0.04^a^(-4.51)	
Server Crowdedness	0.19(1.54)	0.18(1.36)	
Server Competition	0.35^b^(2.39)	0.33^b^(2.20)	
Exchange Rate Change (KRW/USD)	6.34^b^(2.54)		6.65^b^(2.43)
Game Management Company Stock Returns	-1.06^a^(-4.43)		-0.96^a^(-3.42)
Server Fixed Effects are Controlled	Yes	Yes	Yes
Adjusted R^2^	40.7%	36.6%	18.7%
Observations	264	264	264

We examine the determinants of game currency prices with a multivariate regression. Recall Eq 2.

The dependent variable is Log(*Price*_*i*,*t*_), which is the natural log of game currency price in server *i* at week *t*. Independent variables include *Quotes*, *Server Crowdedness*, *Server Competition*, *Exchange Rate*, *and Stock Returns*. *Quotes* is the number of fresh quotes on the market for game currency, *Server Crowdedness* is the number of users in a server measured by a three-step scale (high, medium, and low), *Server Competition* is a dummy variable is equal to one when a server has more competition among players, *Exchange Rate* is the exchange rate between KRW and USD, and *Stock Returns* is the weekly stock returns of the game servicing company. Server fixed effects are controlled. The estimation method is Generalized Method of Moments (GMM) that corrects for heteroscedasticity and autocorrelation in the regression error terms. t-statistics are in parentheses. Coefficients significant at the 1%, 5%, and 10% level are marked by a small a, b, and c, respectively.

Column 1 presents the full model. Column 2 contains a reduced form model that only uses game related variables, while Column 3 reports a model that only uses financial or economic factors. The signs of the coefficients are consistent with our intuition. The demand factors, which are server crowdedness and competition, have positive coefficients. The supply factor, which is the number of professional currency miners, has a negative coefficient. The coefficient on the KRW per one USD exchange rate is positive, as a devaluation of KRW is accompanied with a revaluation of game currency. The coefficients on competition, quotes, and exchange rates are significant at the 5% level. The reduced form models in Columns 2 and 3 demonstrate that the significance of a coefficient does not change much by model specification. The adjusted *R*-square of the full model is 40% indicating that a large portion of the game currency prices is determined by economic factors rather than speculative factors. The reduced form model that only contains financial or economic factors (in Column 3) has an adjusted R-square of 18% in contrast to the almost zero correlation of Bitcoin with other assets. These results demonstrate that the price of some virtual currencies is determined by economic factors.

### Transaction Costs

In this section, we explore another question related to virtual currencies. A major argument for Bitcoin is that the currency can provide lower transaction costs than real currencies, as Bitcoin is free from government regulation/taxation or fees from financial institutions. Theoretically, the P2P (peer-to-peer) transfer system of Bitcoin is free because it is sending data from one user to the other, comparable to sending and receiving emails. In reality, however, often times the P2P system of Bitcoin requires fees for the following reasons. First, fees are necessary to prevent too many small transactions jamming the Bitcoin transaction system. In addition, Bitcoin miners are supposed to use past transaction records when creating additional Bitcoin. A Bitcoin transaction has to be acknowledged by a certain number of Bitcoin miners to become valid. In contrast, miners do not have an obligation to examine and approve transactions. Transaction fees expedite the approval process by rewarding miners. We do not have access to data regarding the level of these “voluntary fees” in the Bitcoin system.

Rather, we focus on the transaction costs of virtual currency exchanges. A large portion of the fees imposed to Bitcoin users actually comes from the exchanges that convert real currencies to Bitcoin. Although the network is growing, Bitcoin is not as widely used as real currencies, so Bitcoin adopters have to convert real currencies to Bitcoin at these exchanges and pay fees. An alternative way of using Bitcoin without paying fees to the exchanges is to mine Bitcoin, but the competition for mining is severe as of 2013, that it was almost impossible to generate enough Bitcoins to do everyday transactions. Bitcoin wiki website (en.bitcoin.it) estimates that as of 2013, it would take approximately five years to mine one Bitcoin with a personal computer. Given that one Bitcoin has a price around $500, buying Bitcoins from exchanges is a much more realistic way of using Bitcoin. In this respect, the math algorithm that controls the supply of Bitcoin acts against the widespread of the currency.

Game currencies share the same characteristics as Bitcoin. In-game transactions with game currencies are P2P transactions and are mostly free of charge. It is often difficult to mine enough currencies from game play alone (although the difficulty is much lower than Bitcoin mining), so players choose to buy game currencies through exchanges that trade game currencies. The exchanges for game currencies have existed since the 1990s indicating that these exchanges are in high demand by virtual currency users.

www.coincompare.com provides a comparison of Bitcoin exchange fees imposed by the exchanges. These fees would include the transaction cost of Bitcoin and the expenses to run a Bitcoin exchange. The Mt. Gox exchange, which handled 70% of the worldwide Bitcoin transactions in 2013, has a fee ranging between 0.25% and 0.60%. Other Bitcoin exchanges charge fees ranging from 0.2% to 2.0%. The only information we have are for game currency exchanges in South Korea, where the exchanges typically impose a 5% fee to sellers only. The fee seems to be much higher than that of Bitcoin exchanges, but only sellers pay the fees and the maximum fee is capped at $30 if the trade size is larger than $600. The sellers of game currencies are mostly professional miners, so most of the retail level exchange users are not directly subject to the fee. Note that in the cases of foreign exchange wire transfers, a retail customer typically pays a wire transfer fee of around $40 per outgoing transfer.

The fee structure of most virtual currency exchanges is not flat or linear, making it difficult to compare one with another. A better method of identifying the true transaction cost is to use various transaction cost estimators endorsed by the market microstructure literature. The literature indicates that the estimators capture various transaction costs, such as the cost of order processing or market making (Stoll [10]). These measures include bid-ask spread, market depth, and sensitivity of prices to trades. Unfortunately, most of the measures are not feasible for virtual currencies as key information, such as the time series data of volume or bid-ask spreads, are often not available. Still, there are two measures that can be derived from only daily prices, which we do have access to. They are the covariance measure of Roll [4] and the Zeros measure of Lesmond et al. [5]. The Roll [4] measure and the Zeros measure have significant correlations with true transaction costs, as demonstrated by Goyenko, Holden, and Trzcinka [11] and Marshall, Nguyen, and Visaltanachoti [12].

The Roll [4] measure calculates the covariance between two consecutive price changes. The measure is intended to capture the price change due to bid-ask spreads (bid-ask bounce). Following Goyenko et al. [11], the Roll [4] measure is defined as:

$$\text{Roll}=\;\{\begin{array}{l} 2\sqrt{-Cov\left(\Delta P_{t},\Delta P_{t-1}\right)}\;When\;Cov\left(\Delta P_{t},\Delta P_{t-1}\right)<0\\ 0\;When\;Cov\left(\Delta P_{t},\Delta P_{t-1}\right)>0 \end{array}$$ (3)

The Zeros measure counts the number of days with zero returns. Many days of zero returns indicates that there is little trading activity, which is associated with higher transaction costs. The Zeros measure is defined as:

$$\text{Zeros}=\left(\frac{\#\;\text{of days with zero returns}}{\text{Number of total trading days}}\right)$$ (4)

We calculate the Roll [4] measure and the Zeros measure of three virtual currencies: two game currencies in our data and Bitcoin. For comparison, the same measures are calculated from the exchange rates of Euro, Japanese Yen (JPY), South Korean Won (KRW), and Chinese Yuan (CNY). Although www.oanda.com reports exchange rates for all weekdays, we find that the returns are frequently zero on Mondays (based on the website’s date). This phenomenon indicates that the Monday rates of this website are recorded when most foreign exchanges are closed. As a response, when calculating the Roll [4] and the Zeros measure from the website’s price data, we omit the observations on Mondays. In addition to real currencies, the measures calculated from U.S. exchange listed equity prices and gold prices are included. If an asset has more than one series in its category, such as small size equities, we report the median of the measure following Marshall et al. [12] as a few large outliers, such as non-trading stocks, may heavily influence the average.

The Roll [4] measure is 7.9% for the *World of Warcraft* currency and 0% for the *Diablo III* currency and Bitcoin. Real currencies have Roll [4] measures ranging from 0% to 0.7%. Among other assets, small size equities have the highest Roll [4] measure of 2.0%. Thus, the Roll [4] measure provides mixed results regarding the hypothesis that virtual currencies have lower transaction costs than real currencies. The Zeros measure displays a similar pattern, but is more positive toward virtual currencies. The highest Zeros measure among virtual currencies is the *World of Warcraft* currency with a Zeros measure of 2.9%. This figure is lower than the Zeros measure of South Korean Won. Bitcoin excels in both measures. The Roll [4] measure is 0% and the Zeros measure is 0.3%. The Zeros measure of Bitcoin is on par with that of the Japanese Yen, which has the lowest Zeros measure among the real currencies reported in Table 4. Overall, we find indications that the transaction costs of virtual currencies are lower than that of real currencies.

**Table 4:** Transaction Costs.

Assets	Roll Measure	Zeros Measure
*World of Warcraft Currencies*	7.2%	2.9%
*Diablo III Currencies*	0.0%	7.4%
*Bitcoin*	0.0%	0.3%
Euro	0.0%	2.6%
Japanese Yen	0.0%	0.3%
South Korean Won	0.8%	3.2%
Chinese Yuan	0.1%	1.6%
Small Equities	2.0%	4.4%
Middle Size Equities	0.8%	2.4%
Large Equities	0.4%	1.2%
Gold	0.0%	6.2%

We calculate the Roll (1984) measure and the Zeros measure of virtual currencies and various assets.

We use the median of each measure by asset class if there are multiple assets in the class, as in the case of small size equities. The data period is from November 2012 to November 2013 for *Diablo III* currency and from July 2013 to November 2013 for *World of Warcraft* currency. We use the full 2013 data for other assets.

### Competition of Exchanges

Bitcoin prices differ by exchange. While in the previous section we use the prices obtained from a Bitcoin exchange that has considerable trading activity and sometimes report a price comparison across different exchanges, the price difference raises a question as to how exchange affects transaction costs. At a glance, one may think that there should not be a huge difference in transaction costs by exchange, as the exchanges are trading the same virtual currency that can be transferred almost freely in cyber space. In reality, however, many of the virtual currency exchanges are not facing high competition because there are barriers preventing traders from switching. Most of these barriers are country specific regulations, such as requiring a trader to have a valid social security number of the country to open an account for an exchange. As we do not have access to Bitcoin historical price data by exchange, we examine this issue by comparing the game currency prices obtained from two exchanges that are located in different countries: the U.S. and South Korea.

While South Korea has relatively large markets for online game currencies, these established markets do not exist in the U.S. Instead, we were able to collect price quotes from a popular game currency dealer in the U.S. The dealer is much smaller in size when compared to exchanges in South Korea. The website posts only their sell quotes, and the sell quotes are updated sporadically (i.e., once a week). The quotes are also much less sophisticated when compared to those of the South Korean market, as the U.S. dealer quotes the same price for all game servers/sides. We have U.S. dealer quotes data for *Diablo III* game currency from May 2012 to June 2012 and for *World of Warcraft* game currency from October 2013 to December 2013.

**Fig 2:**
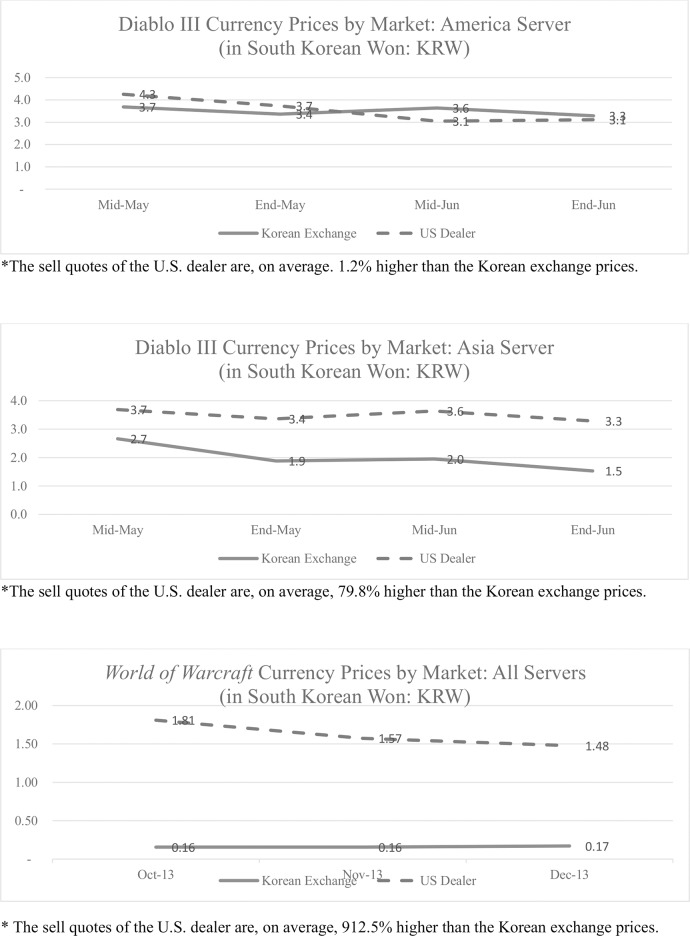
Game Currency Prices Measured from Two Different Countries—in KRW. The game currency prices from two different markets are plotted in this figure. We have approximately two months of sell quotes data from a U.S. based game currency dealer. The quotes are compared with the prices reported by a Korean game currency exchange. The dollar prices of the dealer are converted to South Korean Won using the corresponding exchange rates. *Diablo III* currency prices are from May 2012 to June 2012 and *World of Warcraft* currency prices are from Oct 2013 to Dec 2013.

Since the game currency price of the U.S. dealer website is quoted in U.S. dollars, we convert their sell quotes to South Korean Won (KRW) using the exchange rate between the South Korean Won and the U.S. dollar. We take the average of the *World of Warcraft* currency prices by exchange as there are multiple servers and sides. Fig 2 presents a plot of the prices by exchange. U.S. dealer prices are plotted by dotted lines, and Korean game currency market prices are plotted by solid lines.

In the case of the American server of *Diablo III*, the prices are similar in both exchanges in that the sell quotes of the U.S. dealer are, on average, 1.2% higher than the prices in the Korean market. Since players incur a 5% transaction fee when selling game currency in Korean market, this price difference does not allow an arbitrage transaction when buying game currencies from one market and selling them on another for profit. The prices on the two different markets also move in a similar direction indicating that Purchasing Power Parity somewhat holds for this game currency.

The price difference is larger in the Asian server. The U.S. dealer’s sell quotes are almost twice as high as the Korean exchange prices. This contrast may be because the dealer is operating in the U.S. and they primarily trade on an American server. Consistent with this conjecture, a few months after this sample is acquired, the dealer stopped posting quotes for the Asian server.

The game currency prices of *World of Warcraft* are almost 10 times more expensive in the U.S. A large arbitrage profit would be possible if one can buy game currency in South Korea and sell it in the U.S. This transaction may not be feasible, however, as we only observe sell quotes from the U.S. dealer. The dealer may buy currencies at much lower prices than their sell quotes or impose other restrictions. Note that the dealer is the largest game currency trader in the U.S. thus far, indicating that any game player in the U.S. who wants to buy currencies must contend with these high prices.

The large difference in prices implies that the U.S. dealer is not subject to competition from other exchanges. We wondered why *Diablo III* game currency is relatively well priced on its American server, if U.S. game currency trading does not have much competition. We offer two reasons. First, *Diablo III* has a Real Money Auction House (RMAH) in the game, where players can use real currency to purchase other players’ game items. Players will select RMAH (although there are quite a few restrictions in using the in-game auction house) if the quotes from a game currency dealer are unrealistic. In addition, there are only three servers for *Diablo III*. It will be easier to set a price that ensures profit from all three servers if a dealer quotes one price for all of the servers. *World of Warcraft*, in contrast, has approximately 1,000 servers. This contrast indicates that the transaction costs of virtual currencies can be heavily influenced by the amount of competition among the exchanges. It can be projected that greater competition among exchanges may further diminish the already low transaction costs of Bitcoin and other virtual currencies.

## Summary and Conclusion

This paper examines the price stability and transaction costs of game currencies that have commonalities with Bitcoin. We find that more mature game currencies have a price volatility of one-third of that of Bitcoin, at a level similar to that of small size equities or gold. The decentralized structure of Bitcoin does not seem to be the cause of the recent price instability, as game currencies are also managed by non-government entities. We observe a similar price instability from the game currencies that are launched around the time when Bitcoin gained much of its current public attention (around the year 2011). The contrast between mature and newly introduced virtual currencies indicates that the Bitcoin price may stabilize over time.

The transaction costs of virtual currencies are sometimes lower than that of real currencies. With more competition among virtual currency exchanges, the transaction costs may drop further making virtual currencies a lower cost alternative to real currency transactions. Economists agree that a properly functioning currency should include a method of transaction, a unit of account, and store value (Yermack [3]). Bitcoin may meet the criteria if it can combine its low transaction costs with more stable prices.

However, there are a few caveats for our projection. Bitcoin is the first virtual currency that is attempting to substitute the role of real currencies. Until this point, other virtual currencies, like game currencies, remain as auxiliary currencies that aid in transactions that real currencies cannot easily do, such as transactions within an online game. Game currencies currently have considerable trading volume, but their role is tied to the gaming industry. It is difficult to estimate how widespread Bitcoin will be. Also, our analysis does not justify that virtual currencies should have greater value. A large volume of Bitcoin trading in these days is speculative trading, betting on the possible appreciation of Bitcoin prices. Speculative trades are necessary to discover the reasonable exchange rates of Bitcoin, but it is unknown when the market will reach the equilibrium. As we demonstrate from the comparison of exchanges with varying degrees of competition, various regulations imposed on Bitcoin exchanges may be a dragging factor in the price discovery process.

A note concerning the other uses of game currencies is warranted. The results in this paper suggest that game currencies may be an interesting topic for future research in the areas of monetary economics and consumer behavior. Game currencies are tightly connected to real currencies through the markets for game currencies. Likewise, Castronova [13] finds that game players do not become irrational decision makers because they are in a virtual world. Thus, cyber economies may serve as a lab for monetary policies. For example, economists may examine an ongoing debate as to whether monetary expansion actually creates greater production by gifting each game player with a certain amount of gaming currency. Experiments in this fashion may be much more costly and possibly dangerous in a real economy, while they can be conducted with relative ease in a gaming economy with results that are quantifiable.
